# Adaptive staffing can mitigate essential worker disease and absenteeism in an emerging epidemic

**DOI:** 10.1073/pnas.2105337118

**Published:** 2021-08-16

**Authors:** Elliot Aguilar, Nicholas J. Roberts, Ismail Uluturk, Patrick Kaminski, John W. Barlow, Andreas G. Zori, Laurent Hébert-Dufresne, Benjamin D. Zusman

**Affiliations:** ^a^Division of Gastroenterology, Hepatology and Nutrition, Department of Medicine, University of Florida, Gainesville, FL 32610;; ^b^Vermont Complex Systems Center, University of Vermont, Burlington, VT 05401;; ^c^Department of Electrical Engineering, University of South Florida, Tampa, FL 33612;; ^d^Department of Sociology, Indiana University, Bloomington, IN 47405;; ^e^Center for Complex Networks and Systems Research, Indiana University, Bloomington, IN 47405;; ^f^Department of Animal and Veterinary Sciences, University of Vermont College of Agriculture and Life Sciences, Burlington, VT 05401;; ^g^Department of Computer Science, University of Vermont, Burlington, VT 05401

**Keywords:** COVID-19, pairwise approximation, network model, infectious disease, essential workers

## Abstract

Essential worker absenteeism has been a pressing problem in the COVID-19 pandemic. Nearly 20% of US hospitals experienced staff shortages, exhausting replacement pools and at times requiring COVID-positive healthcare workers to remain at work. To our knowledge there are no data-informed models examining how different staffing strategies affect epidemic dynamics on a network in the context of rising worker absenteeism. Here we develop a susceptible–infected–quarantined-recovered adaptive network model using pair approximations to gauge the effects of worker replacement versus redistribution of work among remaining healthy workers in the early epidemic phase. Parameterized with hospital data, the model exhibits a time-varying trade-off: Worker replacement minimizes peak prevalence in the early phase, while redistribution minimizes final outbreak size. Any “ideal” strategy requires balancing the need to maintain a baseline number of workers against the desire to decrease total number infected. We show that one adaptive strategy—switching from replacement to redistribution at epidemic peak—decreases disease burden by 9.7% and nearly doubles the final fraction of healthy workers compared to pure replacement.

Essential worker absence rates during the COVID-19 pandemic approached 30%, resulting in staff shortages in many sectors, including healthcare (1, 2). Strategies to manage absenteeism typically invoke worker replacement, redistributing work among healthy workers, and recruiting retired workers, despite scant data to assess risk of these interventions (3). The rationale stems from traditional mass-action models, which predict that replacement of sick workers leads to smaller epidemic peaks and sizes. This finding assumes a replacement worker randomly enters an environment of average risk. However, adaptive network models (4) suggest that under certain conditions, replacement workers are at greater risk of infection due to their exchange of relations (i.e., edges) with the sick individuals whom they replace. This “relational exchange” form of replacement can paradoxically result in larger epidemic sizes and accelerated transmission (5). It is unclear how relational exchange and redistribution of work affect epidemic dynamics on a network or under what conditions either one is preferable. To this end, we collected healthcare worker (HCW) contact surveys and absenteeism data from three COVID and non-COVID units in a Florida hospital during the early epidemic. These data inform parameterization of a susceptible–infected–quarantined-recovered pair approximation model. Susceptibles (S) can become infectious (I) at rate βI, with either recovery (R) after an average of 5 d ($1/\gamma_{I}$) or diagnosis at rate ϵ, with subsequent 10-d quarantine (Q) before recovery (6).

In the replacement model, Q individuals are replaced from a staffing pool at compartment-specific rates $r_{S}$, $r_{I}$, $r_{R}$, reflecting local community prevalence (2.97 to 9%) (7). Outgoing workers exchange edges with replacements. In the redistribution model, quarantined worker roles are assumed by remaining workers, without replacement. See *SI Appendix* for more detail.

## Results

**Fig. 1.**
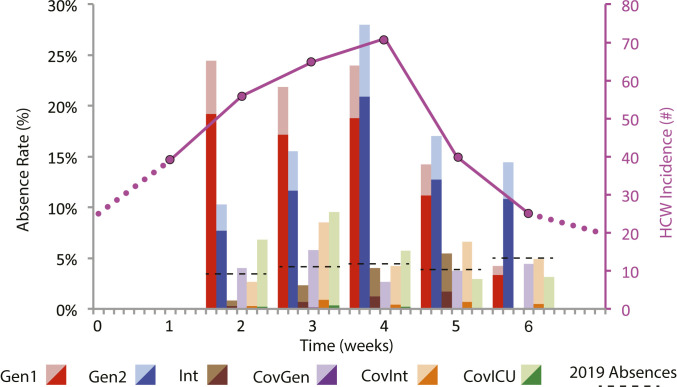
Weekly unit absence percentage (bars) by hospital unit type and total HCW incidence (magenta line). Unit types are Gen, general; Int, intermediate; and ICU, intensive care. COVID-related absences are dark shades. Pre-COVID 2019 absences are black dashed lines. Units with less incidence—counterintuitively COVID (Cov) units—generally had more PPE use (N95 masks) between HCWs, fewer staff shortages, and minor excess absenteeism. Universal surgical mask (but not N95) use and patient (but not HCW) screening were hospital-wide policies. N95 masks and break-room distancing between HCWs were introduced in Gen1 and Gen2 units in the second and the third week, respectively, but were in use from week 0 in Cov units.

HCW absence rates at epidemic peak reached 28% in the non-COVID general units, tracking with HCW incidence (Fig. 1). Only in these two non-COVID units did the COVID-related absence proportion constitute a majority, responsible for upward of 80% of absenteeism. Despite this heterogeneity, relative risk of infection for non-COVID unit HCWs versus COVID unit HCWs was 4.6 (95% CI 2.1 to 10.2), due in part to delayed interventions (e.g., N95 masks) among HCWs in non-COVID units. This is consistent with evidence suggesting that with effective personal protective equipment (PPE), patient–HCW transmission is minimal (8), while less stringent precautions between HCWs can increase the risk for superspreading and asymptomatic transmission (9, 10).

### To Minimize Outbreak Size.

**Fig. 2.**
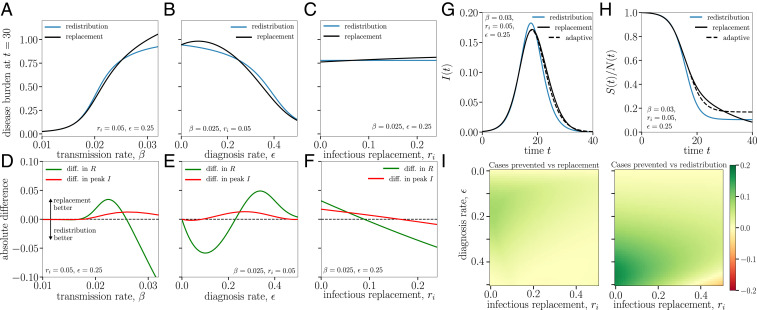
Phenomenology of the replacement and redistribution models, starting from I(0)=0.01. Fixed empirical parameters: average degree $\bar{k}=28$ (*SI Appendix*), $\gamma_{I}=0.2$, $\gamma_{Q}=0.1$, and $r_{r}=0.01$ (6, 7). (*A*–*C*) Disease burden (I+R+Q) after 1 mo as we vary key parameters. No optimal strategy is observed. (*D*–*F*) Trade-offs between the two strategies in minimizing outbreak size (green curve, difference in R at t = 30 d) and peak prevalence [red curve, difference in max[I(t)]]. Population size is normalized to initial workforce but can be >1 because of replacement workers. (*G* and *H*) The difference in temporal spread between workplaces with different strategies, using parameters from *A* and *D* with β=0.03 but initial conditions I(0)=0.1%. An “adaptive” strategy that initially replaces sick workers but switches to redistribution around the epidemic peak (t=17 d) leads to a lower epidemic peak (*G*), while almost doubling the fraction of healthy workers by the end of the outbreak (*H*). (*I*) Heatmaps plot the difference in *H* curves at t = 30 d, comparing the adaptive strategy to replacement (*Left*) and redistribution (*Right*) over a wide range of testing resources and community prevalence. The adaptive strategy leads to more healthy workers in all but the most extreme scenarios ($r_{I}$ and ϵ>0.4).

Our models are parameterized by data shown in Fig. 1 (for disease parameters) and a contact survey administered to 464 HCWs (for network parameters) during the outbreak month. See *SI Appendix* for details and the survey. Fig. 2 compares replacement versus redistribution over a range of β, ϵ, and $r_{I}$, as proxies for PPE use, testing rate and sensitivity, and community prevalence, respectively. If the goal is to minimize outbreak size, neither strategy is clearly preferable across parameter ranges (Fig. 2 *A*–*C*).

For example, to minimize outbreak size at hospital-estimated β and $r_{I}$, redistribution is preferable with a test-and-trace diagnosis approach (ϵ < 0.125). However, as diagnosis rates increase into the realm of frequent screening (ϵ = 0.25, biweekly testing with 89% sensitivity), replacement becomes preferred to minimize outbreak size (Fig. 2 *B* and *E*).

### To Minimize Epidemic Peak.

For the hospital parameterization with biweekly screening (ϵ = 0.25, implying resource-rich capabilities), replacement minimizes epidemic peak when community prevalence $r_{I}$ < 10% (Fig. 2*F*). When community prevalence is >10 to 15%, redistribution is increasingly preferred. At very low ϵ, these two regimes blur, with replacement losing advantage when community prevalence is <10% (Fig. 2*I*).

At higher β, replacement leads to faster early spread by adding fuel to the outbreak (proved analytically in *SI Appendix*, not pictured), but counterintuitively results in lower epidemic peak *I*(*t*) (Fig. 2 *D*, *G*, and *H*).

### Adaptive Strategy and Sensitivity Analysis.

To reap the fruits of replacement in the early phase (lower epidemic peak) and redistribution in the later phase (lower epidemic size), we design a compromise “adaptive” strategy that switches from replacement of workers to redistribution of work just before the epidemic peak (Fig. 2 *G* and *H*, dashed line). The result is a 9.7% decrease in final disease burden (I + R + Q), nearly doubling the fraction of total healthy unaffected workers by the end of the outbreak. Sensitivity analyses for effectiveness (decrease in disease burden) of the adaptive strategy versus replacement and redistribution as a function of diagnosis rate ϵ and community prevalence $r_{I}$ show that the success of the adaptive strategy is minimally sensitive to realistic variations in ϵ and $r_{I}$ (Fig. 2*I*).

## Discussion

During an epidemic, a workplace needs to protect workers from illness (by limiting spread) and avoid absenteeism to preserve a sufficient work force (by decreasing epidemic peak). Our model defines trade-offs in achieving both goals and suggests staffing strategies for resource-poor and resource-rich settings.

While the Centers for Disease Control and Prevention (CDC) and Center for Medicare and Medicaid Services (CMS) generally recommend increased testing rates in response to rising community prevalence for certain locales like long-term care facilities (11), our model shows that the utility of increasing diagnosis rates (ϵ) in a workplace context changes in subtle ways over the course of an outbreak, depending on capability to replace or redistribute staff, varying community prevalence ($r_{I}$), PPE use, and transmission rate (β).

Before the epidemic peak, if the priority is to preserve a baseline work force (e.g., critical infrastructure plants), then replacement of sick workers generally minimizes peak incidence at the expense of more total staff infections (Fig. 2 *G* and *H*).

At the epidemic peak, redistribution generally minimizes epidemic size, while being an inevitable albeit short-term response to severe absenteeism. In a resource-poor setting with limited testing capacity (low ϵ), redistribution is also preferred to limit outbreak size, as it avoids healthy replacements as “fuel” for epidemic spread (Fig. 2 *B*, *E*, and *I*).

While some resource-rich hospitals and institutes of higher education regularly screen all staff, most US hospitals test and trace responsively based on symptoms or exposure, with subsequent quarantine and replacement (12). With regular screening (Fig. 2 *F* and *I*), the preferred strategy hinges on community prevalence ($r_{I}$).

By increasing the likelihood of replacement with an infectious worker, high community prevalence decreases the effectiveness of testing, quarantine, and replacement. This mechanism qualifies general CDC guidelines to test responsively at low prevalence but more regularly as prevalence rises. While responsive testing can be appropriate in resource-poor settings, in resource-rich settings where worker replacement is key (like the hospital parameterization), our results support a strategy of baseline weekly staff screening with an increase to biweekly screening as community prevalence increases from 5 to 10%. This is consistent with CDC’s more aggressive “expanded screening” and CMS long-term care facility guidelines (11), although the specific numerical cutoff is subject to each workplace’s parameterization and resources.

Decisions to replace or redistribute are not mutually exclusive. Redistribution as a policy clearly has limits, for safety and mental health reasons. Yet unique skill sets also limit replacement in many essential worker settings like healthcare. Our model is a first step toward recognizing that replacement of a sick worker with a healthy worker is not necessarily the de facto preference.

The model demonstrates that the optimal staffing strategy depends on the stage of the epidemic and that it is possible to adaptively “tune” between strategies to minimize peak prevalence in the early phase (through worker replacement) and minimize outbreak size afterward (through redistribution of work). This trade-off between epidemic peak and size has not been adequately considered in theory or practice. How this trade-off translates into policy prescription is complex and merits a cost–benefit analysis beyond our scope. While the adaptive staffing strategy’s incremental benefits are substantial, its incremental costs are challenging to valuate across essential work sectors as varied as healthcare, manufacturing, and agriculture. In light of these uncertainties and our stylized model without explicit heterogeneity, specific staffing recommendations should be interpreted with caution, particularly when extrapolating to different workplaces.

More extensive models are needed before using these results as a basis to allocate resources. With appropriate parameterization, the model can apply to a variety of workplaces. In the context of a resource-rich hospital, a potential improved staffing strategy is early phase replacement with vigorous testing, followed by temporary redistribution of work at epidemic peak. Since absenteeism can reach 30% with minor increases in workplace incidence, even small gains in minimizing outbreak size can be significant in mitigating staff shortages and disease burden, especially given recent, more transmissible COVID-19 variants (13).

## Materials and Methods

HCW mean degree $\bar{k}=28$ in our contact survey. HCW absences in Fig. 1 track unit-based employees, including nurses, patient care assistants, nurse managers, and support technicians (Datasets S1 and S2).

Flow between states and parameters are described in the main text and *SI Appendix*. Replacement rates for each compartment $r_{S}$, $r_{I}$, $r_{R}$ are parameterized from local data (7). Diagnosis rate ϵ = (testing rate × test sensitivity), with values in main text and Fig. 2.

Absence is operationalized as a missed work shift for unplanned reasons, with absence rate = (total absent shifts)/(total worked shifts). COVID-related absences, expressed as percentage of total missed shifts, include those secondary to diagnosis or pending test results.

All cases were diagnosed by RT-PCR nasal swab. The University of Florida institutional review board approved the study (IRB202001069) with documentation of informed consent waived.

## Supplementary Material

Supplementary File

Supplementary File

Supplementary File

## Data Availability

Derivations and data are included in the article and *SI Appendix*.
