# Supplementary material for: Adaptive staffing can mitigate essential worker disease and absenteeism in an emerging epidemic
Source: Proc Natl Acad Sci U S A. 2021 Aug 16;118(34):e2105337118. doi: 10.1073/pnas.2105337118 (PMC8403949; doi:10.1073/pnas.2105337118)
Supplement: Supplementary File [file pnas.2105337118.sapp.pdf]

# Supplementary Information for

## Adaptive staffing can mitigate essential worker disease and absenteeism in an emerging epidemic

**Elliot Aguilar, Nicholas Roberts, Ismail Uluturk, Patrick Kaminski, John W Barlow, Andreas G. Zori, Laurent Hébert-Dufresne, Benjamin D. Zusman**

**Benjamin D. Zusman.**
**E-mail: benjamin.zusman@medicine.ufl.edu**

### This PDF file includes:

Supplementary text
Legends for Dataset S1 to S2
SI References

### Other supplementary materials for this manuscript include the following:

Datasets S1 to S2

## Supporting Information Text

### Extended Methods.

***Replacement Model.*** We model a network-structured population in which infectious individuals are diagnosed and sequestered (i.e. sent to the $Q$ compartment), whence they recover with rate $\gamma_Q$, and return to the population. We assume that the working population, which consists of susceptibles ($S$), infectious ($I$) individuals that have not yet been diagnosed, and recovered ($R$) individuals, must remain constant in time. Individuals are discovered to be infectious (i.e. 'diagnosed') with rate $\epsilon$. Infectious individuals recover directly with rate $\gamma_I$. In order to maintain essential roles, individuals removed for quarantine are replaced from an external reservoir of individuals who 'inherit' their network connections. This replacement individual is in one of the three disease states $S$,$I$,$R$, with rates $r_S$,$r_I$,$r_R$, respectively, which can be determined by population rates of prevalence of the infection.

The rates of change of expected number of individuals in each compartment is governed by the following system of equations:

$$
\begin{aligned}
\frac{d}{dt}[S] &= -\beta[SI] + r_S(\epsilon[I] - \gamma_Q[Q]) && [1]\\
\frac{d}{dt}[I] &= \beta[SI] - \epsilon[I] - \gamma_I[I] + r_I(\epsilon[I] - \gamma_Q[Q]) && [2]\\
\frac{d}{dt}[R] &= \gamma_I[I] + \gamma_Q[Q] + r_R(\epsilon[I] - \gamma_Q[Q]) && [3]\\
\frac{d}{dt}[Q] &= \epsilon[I] - \gamma_Q[Q] && [4]\\
& && [5]
\end{aligned}
$$

Importantly, the quantities in brackets are expected counts, not densities, as the overall system is not constrained in size (due to recruitment from the population reservoir). The system above induces the following system of pair equations:

$$
\begin{aligned}
\frac{d}{dt}[SI] &= \beta[SSI] - 2\beta[ISI] - \beta[SI] - \gamma_I[SI] + 2r_S\epsilon[II] - r_S\gamma_Q\frac{[Q]}{[S]}[SI] - r_I\gamma_Q\frac{[Q]}{[I]}[SI] - r_S\epsilon[SI] - r_R\epsilon[SI]\\
\frac{d}{dt}[SS] &= r_S\epsilon[SI] - 2r_S\gamma_Q\frac{[Q]}{[S]}[SS] - \beta[SSI]\\
\frac{d}{dt}[SR] &= \gamma_I[SI] + r_S\epsilon[IR] + r_R\epsilon[SI] - r_S\gamma_Q\frac{[Q]}{[S]}[SR] - \beta[ISR] + 2r_S\gamma_Q\frac{[Q]}{[S]}[SS] + r_I\gamma_Q\frac{[Q]}{[I]}[SI]\\
\frac{d}{dt}[IR] &= \beta[ISR] + 2\gamma_I[II] + 2r_R\epsilon[II] - r_R\epsilon[IR] - \gamma_I[IR] - r_S\epsilon[IR] + r_S\gamma_Q\frac{[Q]}{[S]}[SI] + 2r_I\gamma_Q\frac{[Q]}{[I]}[II] - r_I\gamma_Q\frac{[Q]}{[I]}[IR]\\
\frac{d}{dt}[II] &= \beta[SI] - 2(r_S + r_R)\epsilon[II] - 2r_I\gamma_Q\frac{[Q]}{[I]}[II] - 2\gamma_I[II] + 2\beta[ISI]\\
\frac{d}{dt}[RR] &= r_S\gamma_Q\frac{[Q]}{[S]}[SR] + \gamma_I[IR] + r_R\epsilon[IR] + r_I\gamma_Q\frac{[Q]}{[I]}[IR]
\end{aligned}
$$

We complete pair approximations by assuming the same triple closures as (1), where $\overline{k}$ is the expected degree of a node in the network, and $\overline{q} = \overline{k^2}/\overline{k}$ is the expected degree of a neighbor,

$$
\begin{aligned}
\frac{d}{dt}[SI] &= 2\beta\frac{(\bar{q}-1)}{\bar{k}}[SS]\frac{[SI]}{[S]} - \beta\frac{(\bar{q}-1)}{\bar{k}}\frac{[SI]^2}{[S]} - \left(\beta + \gamma_I + \epsilon(r_S + r_R) + r_S\gamma_Q\frac{[Q]}{[S]} + r_I\gamma_Q\frac{[Q]}{[I]}\right)[SI] + 2r_S\epsilon[II]\\
\frac{d}{dt}[SS] &= -2\beta\frac{(\bar{q}-1)}{\bar{k}}[SS]\frac{[SI]}{[S]} - 2r_S\gamma_Q\frac{[Q]}{[S]}[SS] + (r_S\epsilon)[SI]\\
\frac{d}{dt}[SR] &= -\beta\frac{(\bar{q}-1)}{\bar{k}}[SI]\frac{[SR]}{[S]} + r_S\epsilon[IR] - r_S\gamma_Q\frac{[Q]}{[S]}[SR] + 2r_S\gamma_Q\frac{[Q]}{[S]}[SS] + (\gamma_I + r_R\epsilon)[SI] + r_I\gamma_Q\frac{[Q]}{[I]}[SI]\\
\frac{d}{dt}[IR] &= \beta\frac{(\bar{q}-1)}{\bar{k}}[SI]\frac{[SR]}{[S]} + 2\left(\gamma_I + r_R\epsilon + r_I\gamma_Q\frac{[Q]}{[I]}\right)[II] - (r_R\epsilon + \gamma_I + r_S\epsilon)[IR] + r_S\gamma_Q\frac{[Q]}{[S]}[SI] - r_I\gamma_Q\frac{[Q]}{[I]}[IR]\\
\frac{d}{dt}[II] &= \beta\frac{(\bar{q}-1)}{\bar{k}}\frac{[SI]^2}{[S]} + \beta[SI] - 2\left(\epsilon(r_S + r_R) + \gamma_I\right)[II]\\
\frac{d}{dt}[RR] &= r_S\gamma_Q\frac{[Q]}{[S]}[SR] + \gamma_I[IR] + r_R\epsilon[IR] + r_I\gamma_Q\frac{[Q]}{[I]}[IR].
\end{aligned}
$$

Following (2), we define

$$
\mathcal{C}_{SI} = \frac{N}{\bar{k}}\frac{[SI]}{[S][I]}, \qquad [6]
$$

a measure of the correlation in $SI$ pairs. When $\mathcal{C}_{SI} = 1$, $SI$ pairs are formed purely at random; for any $\mathcal{C}_{SI}$ greater than 1, $S$ and $I$ individuals are more likely than random to be paired.

Using this measure of the correlation, we can rewrite the equation for $d[I]/dt$,

$$\frac{d}{dt}[I] = \beta\frac{[S][I]\overline{k}}{N}\mathcal{C}_{SI} - (\epsilon + \gamma_I - r_I\epsilon)[I] - r_I\gamma_Q[Q]. \quad [7]$$

We then can solve for $R_0$,

$$R_0 = \frac{\beta\overline{k}}{\epsilon(r_S + r_R) + \gamma_I}\mathcal{C}_{SI}, \quad [8]$$

where, as pointed out by (2), we must consider the quasi-equilibrium value, $\mathcal{C}^*_{SI}$, that forms in the early period of the epidemic. Importantly, $R_0$ grows as $\mathcal{C}^*_{SI}$, which again measures the correlation in $SI$ pairs. Examining the 'decay' of $\mathcal{C}_{SI}$ in the early period of the epidemic,

$$\frac{d}{dt}\mathcal{C}_{SI} \quad = \quad \frac{N}{\overline{k}}\frac{d}{dt}\left(\frac{[SI]}{[S][I]}\right) \to \beta(\overline{q}-2)\mathcal{C}_{SI} + r_S\epsilon\frac{[I]}{[S]}\mathcal{C}_{\mathcal{II}} - \beta\overline{k}\mathcal{C}^2_{SI} \quad [9]$$

as $[S] \to N$, $[I] \to 1$, $[Q] \to 0$. We can see that the quasi-equilibrium value of $\mathcal{C}^*_{SI}$ depends on $\frac{[I]}{[S]}\mathcal{C}_{\mathcal{II}}$, (note: while $\frac{[I]}{[S]} \to 0$ in the limit, $\mathcal{C}_{\mathcal{II}} \to \infty$). Considering the decay of this term,

$$\frac{d}{dt}\left(\frac{[I]}{[S]}\mathcal{C}_{\mathcal{II}}\right) = \frac{2N}{\overline{k}}\frac{d}{dt}\left(\frac{[II]}{[S][I]}\right) \to \frac{[I]}{[S]}\mathcal{C}_{\mathcal{II}}(\beta\overline{k} + \epsilon(r_S + r_R) + \gamma_I) + 2\beta\mathcal{C}_{SI} \quad [10]$$

and thus,

$$\frac{[I]}{[S]}\mathcal{C}_{\mathcal{II}} \to \frac{2\beta\mathcal{C}_{SI}}{\beta\overline{k}\mathcal{C}_{SI} + \epsilon(r_S + r_R) + \gamma_I}. \quad [11]$$

Substituting this value into eq. 10, we see that $\mathcal{C}^*_{SI}$ must satisfy,

$$\beta(\overline{q}-2)\mathcal{C}^*_{SI} - \beta\overline{k}\mathcal{C}^{*2}_{SI} + \frac{2\beta\mathcal{C}^*_{SI}}{\beta\overline{k}\mathcal{C}^*_{SI} + \epsilon(r_S + r_R) + \gamma_I} = 0. \quad [12]$$

***Redistribution Model.*** Again, we model a network-structured population in which infectious individuals are diagnosed, with rate $\epsilon$, and sequestered (i.e. sent to $Q$), where they recover with rate $\gamma_Q$, and return to the population. In the current model, sequestered individuals are not replaced; instead their network edges are reassigned to non-sequestered individuals at random.

The model leads to the following system of compartmental equations,

$$\begin{aligned}
\frac{d}{dt}[S] &= -\beta[SI] \\
\frac{d}{dt}[I] &= \beta[SI] - (\epsilon + \gamma_I)[I] \\
\frac{d}{dt}[R] &= \gamma_Q[Q] + \gamma_I[I] \\
\frac{d}{dt}[Q] &= \epsilon[I] - \gamma_Q[Q]
\end{aligned}$$

We then have the following system of pair equations:

$$
\begin{aligned}
\frac{d}{dt}[SI] &= \beta[SSI] - 2\beta[ISI] - \beta[SI] - \gamma_{I}[SI] - \epsilon[SI]\frac{[S]+[R]}{[S]+[I]+[R]} \\
&\quad -\gamma_q[Q]\bar{k}\frac{[SI]}{[SI]+[SS]+[SR]+[IR]+[II]+[RR]} \\
\frac{d}{dt}[SS] &= -\beta[SSI] + \epsilon[SI]\frac{[S]}{[S]+[I]+[R]} - \gamma_q[Q]\bar{k}\frac{[SS]}{[SI]+[SS]+[SR]+[IR]+[II]+[RR]} \\
\frac{d}{dt}[SR] &= \gamma_{I}[SI] - \beta[ISR] + \epsilon\left([SI]\frac{[R]}{[S]+[I]+[R]} + [IR]\frac{[S]}{[S]+[I]+[R]}\right) \\
&\quad +\gamma_q[Q]\bar{k}\left(\frac{[S]}{[S]+[I]+[R]} - \frac{[SR]}{[SI]+[SS]+[SR]+[IR]+[II]+[RR]}\right) \\
\frac{d}{dt}[IR] &= \beta[ISR] + 2\gamma_{I}[II] - \gamma_{I}[IR] + \epsilon\left(2[II]\frac{[R]}{[S]+[I]+[R]} - [IR]\frac{[S]+[R]}{[S]+[I]+[R]}\right) \\
&\quad +\gamma_q[Q]\bar{k}\left(\frac{[I]}{[S]+[I]+[R]} - \frac{[IR]}{[SI]+[SS]+[SR]+[IR]+[II]+[RR]}\right) \\
\frac{d}{dt}[II] &= \beta[SI] + 2\beta[ISI] - 2\left(\epsilon\frac{[S]+[R]}{[S]+[I]+[R]} + \gamma_{I}\right)[II] - \gamma_q[Q]\bar{k}\frac{[II]}{[SI]+[SS]+[SR]+[IR]+[II]+[RR]} \\
\frac{d}{dt}[RR] &= \gamma_{I}[IR] + \epsilon[IR]\frac{[R]}{[S]+[I]+[R]} + \gamma_q[Q]\bar{k}\left(\frac{[R]}{[S]+[I]+[R]} - \frac{[RR]}{[SI]+[SS]+[SR]+[IR]+[II]+[RR]}\right) \\
\frac{d}{dt}\bar{k} &= \frac{-2([SI]+[SS]+[SR]+[IR]+[II]+[RR]) * (\frac{d}{dt}[S] + \frac{d}{dt}[I] + \frac{d}{dt}[R])}{([S]+[I]+[R])^2}
\end{aligned}
$$

After applying the triple closures, we can re-express the above as,

$$
\begin{aligned}
\frac{d}{dt}[SI] &= 2\beta\frac{(\overline{q}-1)}{\bar{k}}[SS]\frac{[SI]}{[S]} - \beta\frac{(\overline{q}-1)}{\bar{k}}\frac{[SI]^2}{[S]} - \beta[SI] - \gamma_{I}[SI] - \epsilon[SI]\frac{[S]+[R]}{[S]+[I]+[R]} + 2\epsilon[II]\frac{[S]}{([S]+[I]+[R])} \\
&\quad -\gamma_q[Q]\bar{k}\frac{[SI]}{[SI]+[SS]+[SR]+[IR]+[II]+[RR]} \\
\frac{d}{dt}[SS] &= -2\beta\frac{(\overline{q}-1)}{\bar{k}}[SS]\frac{[SI]}{[S]} + \epsilon[SI]\frac{[S]}{[S]+[I]+[R]} - \gamma_q[Q]\bar{k}\frac{[SS]}{[SI]+[SS]+[SR]+[IR]+[II]+[RR]} \\
\frac{d}{dt}[SR] &= \gamma_{I}[SI] - \beta\frac{(\overline{q}-1)}{\bar{k}}[SI]\frac{[SR]}{[S]} + \epsilon\left([SI]\frac{[R]}{[S]+[I]+[R]} + [IR]\frac{[S]}{[S]+[I]+[R]}\right) \\
&\quad +\gamma_q[Q]\bar{k}\left(\frac{[S]}{[S]+[I]+[R]} - \frac{[SR]}{[SI]+[SS]+[SR]+[IR]+[II]+[RR]}\right) \\
\frac{d}{dt}[IR] &= \beta\frac{(\overline{q}-1)}{\bar{k}}[SI]\frac{[SR]}{[S]} + 2\gamma_{I}[II] - \gamma_{I}[IR] + \epsilon\left(2[II]\frac{[R]}{[S]+[I]+[R]} - [IR]\frac{[S]+[R]}{[S]+[I]+[R]}\right) \\
&\quad +\gamma_q[Q]\bar{k}\left(\frac{[I]}{[S]+[I]+[R]} - \frac{[IR]}{[SI]+[SS]+[SR]+[IR]+[II]+[RR]}\right) \\
\frac{d}{dt}[II] &= \beta[SI] + \beta\frac{(\overline{q}-1)}{\bar{k}}\frac{[SI]^2}{[S]} - 2\left(\epsilon\frac{[S]+[R]}{[S]+[I]+[R]} + \gamma_{I}\right)[II] - \gamma_q[Q]\bar{k}\frac{[II]}{[SI]+[SS]+[SR]+[IR]+[II]+[RR]} \\
\frac{d}{dt}[RR] &= \gamma_{I}[IR] + \epsilon[IR]\frac{[R]}{[S]+[I]+[R]} + \gamma_q[Q]\bar{k}\left(\frac{[R]}{[S]+[I]+[R]} - \frac{[RR]}{[SI]+[SS]+[SR]+[IR]+[II]+[RR]}\right) \\
\frac{d}{dt}\bar{k} &= \frac{-2([SI]+[SS]+[SR]+[IR]+[II]+[RR]) * (\frac{d}{dt}[S] + \frac{d}{dt}[I] + \frac{d}{dt}[R])}{([S]+[I]+[R])^2}
\end{aligned}
$$

$R_0$ is given by,

$$R_0 = \frac{\beta\overline{k}}{\epsilon + \gamma_{I}}\mathcal{C}_{SI}. \quad [13]$$

Using the same method as above, we have,

$$\frac{d}{dt}\mathcal{C}_{SI} \rightarrow \beta(\overline{q}-2)\mathcal{C}_{SI} + \epsilon\frac{[I]}{[S]}\mathcal{C}_{II} - \beta\overline{k}\mathcal{C}_{SI}^2, \quad [14]$$

where again we have a dependency on the $\epsilon\frac{[I]}{[S]}\mathcal{C}_{II}$ term. In the limit,

$$\frac{[I]}{[S]}\mathcal{C}_{II} \rightarrow \frac{2\beta\mathcal{C}_{SI}}{\beta\overline{k}\mathcal{C}_{SI} + \gamma_I + \epsilon}. \quad [15]$$

Thus, our quasi-equilibrium value, $\mathcal{C}^*_{SI}$, must satisfy the following equation:

$$\beta(\overline{q} - 2)\mathcal{C}^*_{SI} + \frac{2\epsilon\beta\mathcal{C}_{SI}}{\beta\overline{k}\mathcal{C}_{SI} + \gamma_I + \epsilon} - \beta\overline{k}\mathcal{C}^{*2}_{SI} = 0. \quad [16]$$

***Comparison of*** $R_0$**.** Let $R_0^{Rep.}$ be the $R_0$ value for the replacement model, and $R_0^{Red.}$ be the $R_0$ value for the redistribution model. We wish the know when $R_0^{Rep.} > R_0^{Red.}$.

In both models, $R_0$ depends on the quasi-equilibrium value $C^*_{SI}$, which satisfies conditions 12 and 16 for the replacement and redistribution models, respectively. Both of these conditions can be re-expressed as quadratic equations in $C^*_{SI}$. For the redistribution model, the coefficients of this quadratic are:

$$\begin{aligned} a_{red.} &= -\beta^2\overline{k}^2 \\ b_{red.} &= \beta\overline{k}[\beta(\overline{q} - 2) - (\epsilon + \gamma_I)] \\ c_{red.} &= \beta[(\overline{q} - 2)(\epsilon + \gamma_I) + 2\epsilon] \end{aligned} \quad [17]$$

and similarly for the replacement model:

$$\begin{aligned} a_{rep.} &= -\beta^2\overline{k}^2 \\ b_{rep.} &= \beta\overline{k}[\beta(\overline{q} - 2) - (\epsilon(r_S + r_R) + \gamma_I)] \\ c_{rep.} &= \beta[(\overline{q} - 2)(\epsilon(r_S + r_R) + \gamma_I) + 2r_S\epsilon] \end{aligned} \quad [18]$$

As the parameters, $\beta, \overline{k}, \overline{q}, \epsilon, r_S, r_I, r_R, \gamma_I > 0$, it is easy to show that,

$$|b_{rep.}| \quad > \quad |b_{red.}|, \quad [19]$$

$$c_{rep.} \quad < \quad c_{red.}. \quad [20]$$

The above conditions allow us to re-express the inequality, $R_0^{Rep.} > R_0^{Red.}$, as

$$\frac{b_{rep.} + \sqrt{b^2_{rep.} - 4a_{rep.}c_{rep.}}}{\beta\overline{k}(\epsilon(r_S + r_R) + \gamma_I)} > \frac{b^2_{red.} + \sqrt{b^2_{red.} - 4a_{red.}c_{red.}}}{\beta\overline{k}(\epsilon + \gamma_I)}. \quad [21]$$

When $\overline{q} > 2$ (for a Poisson network, this is equivalent to $\overline{k} > 1$ as $\overline{q} = \overline{k} + 1$),

$$R_0^{Rep.} > R_0^{Red.} \Rightarrow 1 < \frac{(\epsilon + \gamma_I)\sqrt{b^2_{rep.} - 4a_{rep.}c_{rep.}}}{(\epsilon(r_S + r_R) + \gamma_I)\sqrt{b^2_{red.} - 4a_{red.}c_{red.}}}. \quad [22]$$

For the parameter regions considered in Fig. 2 in the paper, the preceding condition holds and $R_0^{Rep.} > R_0^{Red.}$.

## Legends for Dataset S1 to S3

### SI Dataset S1 (DatasetS1.xlsx)

**Health care worker (HCW) survey:** The hospital is an approximately 1000-bed, academic, tertiary care center. We administered a cross sectional egocentric survey of HCWs via RedCap to assess contact networks and demographic characteristics. Inclusion criterion was all hospital staff present since the first local Covid-19 case. An estimated total of 4572 surveys were distributed by email via departmental champions, and 583 surveys were submitted, of which 464 were valid after exclusion of those who did not include job type or whose answers were non-interpretable. The effective response rate was 10%, representing approximately 5% of the total HCW population. Administrative workers were poorly sampled (4 total). HCW types are defined by role rather than title, grouping together those with similar duties and contact patterns. For example, "Nurse" includes largely *unit-based* registered nurses, licensed practical nurses, nurse aids, patient care assistants, and support technicians. The complete survey is included in the second tab of this spreadsheet; it includes more extensive questions, responses to which are available with appropriate data sharing agreement per IRB approval.

### SI Dataset S2 (DatasetS2.xlsx)

**HCW Absenteeism and Covid-19 Incidence:** Outcome variables were number of absences by day, unit, and HCW type; Covid-19 related absence proportion; and total incidence. Data was aggregated by week. The weekly incidence curve in Fig. 1 is inclusive of the whole hospital. Hospital absences are recorded as UTO (Unpaid Time Off) and PTO (Paid Time Off) shifts missed. Covid work-related illness is considered PTO, so it has been manually added to the UTO count to calculate total number missed shifts. We use UTO/PTO as a baseline operationalized measure of absenteeism, choosing not to make assumptions about the average number of UTOs per Covid case (on average 1-2) or number of shifts missed per UTO (on average 1-3). As such, our absence rates are a lower bound of the traditional Employee Absence Rate, which counts "Days Absent" in the denominator instead of UTO and PTOs. Baseline (weighted average) weekly absenteeism across the 6 units for the same month in 2019 ranged from 3-5%, with an average of 4.2%, expressed by the dotted line in Fig. 1.

## References


1. C Llensa, D Juher, J Saldana, On the early epidemic dynamics for pairwise models. *J. theoretical biology* **352**, 71–81 (2014).
2. MJ Keeling, The effects of local spatial structure on epidemiological invasions. *Proc. Royal Soc. London. Ser. B: Biol. Sci.* **266**, 859–867 (1999).
